# Quercetin Ameliorates Insulin Resistance and Restores Gut Microbiome in Mice on High-Fat Diets

**DOI:** 10.3390/antiox10081251

**Published:** 2021-08-05

**Authors:** Yuqing Tan, Christina C. Tam, Matt Rolston, Priscila Alves, Ling Chen, Shi Meng, Hui Hong, Sam K. C. Chang, Wallace Yokoyama

**Affiliations:** 1 Beijing Laboratory for Food Quality and Safety, College of Food Science and Nutritional Engineering, China Agriculture University, Beijing 100083, China; yuqingtan@cau.edu.cn; 2 Healthy Processed Foods Research Unit, Agricultural Research Service, United States Department of Agriculture, Albany, CA 94710, USA; priscila.alves@usda.gov (P.A.); lingchen@jiangnan.edu.cn (L.C.); wally.yokoyama@usda.gov (W.Y.); 3 Foodborne Toxins Detection and Prevention Research Unit, Agricultural Research Service, United States Department of Agriculture, Albany, CA 94710, USA; christina.tam@usda.gov; 4 Host Microbe Systems Biology Core, University of California, One Shields Avenue, Davis, CA 95616, USA; mrrolston@ucdavis.edu; 5 School of Food Science and Technology, Jiangnan University, Wuxi 214122, China; 6 Nestlé R & D (China) Ltd., Beijing 100015, China; 7 Key Research Laboratory of Agro-Products Processing, Institute of Food Science and Technology, Chinese Academy of Agricultural Sciences, Beijing 100193, China; 8 Experimental Seafood Processing Laboratory, Costal Research and Extension Center, Mississippi State University, Biloxi, MS 39579, USA; sc1690@msstate.edu

**Keywords:** quercetin, high-fat diet, insulin resistance, gut microbiome

## Abstract

Quercetin is a flavonoid that has been shown to have health-promoting capacities due to its potent antioxidant activity. However, the effect of chronic intake of quercetin on the gut microbiome and diabetes-related biomarkers remains unclear. Male C57BL/6J mice were fed HF or HF supplemented with 0.05% quercetin (HFQ) for 6 weeks. Diabetes-related biomarkers in blood were determined in mice fed high-fat (HF) diets supplemented with quercetin. Mice fed the HFQ diet gained less body, liver, and adipose weight, while liver lipid and blood glucose levels were also lowered. Diabetes-related plasma biomarkers insulin, leptin, resistin, and glucagon were significantly reduced by quercetin supplementation. In feces, quercetin supplementation significantly increased the relative abundance of *Akkermansia* and decreased the Firmicutes/Bacteroidetes ratio. The expression of genes *Srebf1*, *Ppara*, *Cyp51*, *Scd1*, and *Fasn* was downregulated by quercetin supplementation. These results indicated that diabetes biomarkers are associated with early metabolic changes accompanying obesity, and quercetin may ameliorate insulin resistance.

## 1. Introduction

Obesity is recognized as a major global public health crisis. In 2016, over 1.9 billion adults globally were overweight and 650 million were obese globally [1]. Obesity, cardiovascular disease, and type II diabetes are considered inflammatory diseases. Anti-inflammatory phytochemicals such as phenolics and polyphenolics are extremely potent against metabolic diseases. They are concentrated in leaves, peels, and seeds where they protect the plant against environmental pathogens. Their bioactivity against plant pathogens is broad and may be the basis for their demonstrated beneficial health properties in humans.

Quercetin is one of the most abundant and common flavonoids in plant foods [2]. It is well known as a potent antioxidant and scavenger of reactive oxygen species (ROS) and reactive nitrogen species (RNS). Quercetin has been shown to have beneficial effects in human studies [3]. However, the effect of chronic intake of quercetin on the gut microbiome and diabetes-related biomarkers remains unclear. However, quercetin content in typical meals for humans does not reach the high levels used in some animal studies. Therefore, understanding the effect of a chronic intake of a low concentration (0.05%) of quercetin on the gut microbiome in mice on a high-fat diet is vital. Quercetin is found as a glycoside in foods, and studies suggest that the glycoside must be hydrolyzed to the aglycone for efficient absorption [4]. The pharmacological effects of quercetin may also be partly due to its physicochemical properties. The solubility of quercetin is low, about 2.6 mg/L at 25 °C. Despite its low solubility, carbon-14 (^14^C) studies in humans reported that oral absorption was 36–53%. However, the combined urinary and fecal excretion was less than 10% [5]. The investigators found that as much as 23–81% was excreted as CO_2_, suggesting that microbial action may contribute to its degradation. A recent study suggested that the metabolism of the natural product asperuloside altered intestinal metabolites levels and composition via modulation of gut microbiota [6].

Quercetin has been shown to be highly effective in preventing obesity-related metabolic syndrome characteristics in animals. For example, quercetin (0.05%) reduced body weight, visceral fat, blood glucose, insulin, and TNF-α in C57BL/6J mice fed a high-fat diet for 20 weeks but not 8 weeks. However, this study did not investigate the gut microbiome [7,8]. In terms of a low-fat diet, no significant effects on body weight, visceral fat, or blood glucose and lipids were observed in C57BL/6J mice fed 0.05% or 1% quercetin in an AIN93G diet (7% fat by weight) for 20 weeks [9]. However, type 2 diabetic mice (db/db mice) fed 0.04% or 0.08% quercetin on a AIN93G diet for 6 weeks had lower fasting glucose but not insulin, as well as lower adiponectin at 0.08% but not 0.04% quercetin [10]. These results suggest that quercetin reduces biomarkers of metabolic dysfunction when mice have excessive body and visceral adipose weight gain via consumption of a high ft diet or in an obesogenic animal model.

Quercetin also changes the distribution of gut bacteria. In overweight humans and animals, the intake of probiotics and dietary fibers changed the patterns of gut microbiota in comparison to those of normal-weight animals or humans [11]. Gut bacteria transform quercetin to metabolites such as homoprocatechuic acid, protocatechuic acid, 4-hydroxybenzoic acid, and propionic acid [12]. These metabolites can be detected in blood and urine to assess bioactivity in human trials [13]. Quercetin (1% of diet) was reported to reshape the fecal microbiota composition of rats fed a high-fat, sucrose diet, but anti-obesity and anti-inflammation effects were not reported [14]. However, 1% quercetin supplementation is hard to achieve in typical meals for humans (usual consumption is 10–100 mg quercetin a day). While many studies have investigated the impact of quercetin intake on physiological effects including changes in gene expression and, levels of lipid- and carbohydrate-metabolizing enzymes, few have reported alterations in biomarkers associated with insulin resistance (IR) and appetite. This is the first study to investigate chronic intake and its effect on gut microbiota profiles in diet-induced insulin-resistant mice. We hypothesize that the prevention of insulin resistance and obesity, as well as improvements in the gut microbiome, due to quercetin intake in diet-induced obese mice is accompanied by measurable changes in blood biomarkers for diabetes.

## 2. Materials and Methods

### 2.1. Animal and Diets

Male C57BL/6J mice (22.3 ± 1.5 g), 5 weeks old, were purchased from Jackson Laboratories (Sacramento, CA, USA). Mice were housed individually in a temperature-controlled room (20–22 °C, 60% relative humidity, 12 h alternating light/dark cycle). Mice were acclimated and given access to drinking water and chow diet (LabDiet #5001, PMI International, Redwood, CA, USA) *ad libitum* for 1 week before feeding of the experimental diet. Mice were sorted by weight, and each weight range was randomly divided into three groups with eight mice each and fed, ad libitum, semi-synthetic diets based on an AIN-93G formulation consisting of a high-fat (HF, 53% fat calories) diet supplemented with 0.05% quercetin (HFQ), an HF control diet, and a low-fat (LF) reference diet (Table 1). Quercetin was dissolved in 5 mL of ethanol dispersed with the dry ingredients, followed by evaporation of ethanol to obtain 1 kg of food. Body weights were recorded once a week, and food intake was monitored twice a week. The study was reviewed and approved by the Institutional Animal Care and Use Committee, Western Regional Research Center, USDA, Albany, CA, USA (Protocol No. 18-4).

### 2.2. Plasma and Tissue Collection

After 6 weeks of feeding, mice were fasted for 16 h and then anesthetized with isoflurane (Phoenix Pharmaceutical, St. Joseph, MO, USA). Blood was collected by cardiac puncture into EDTA-rinsed syringes. Plasma was separated by centrifugation at 2000× *g* for 15 min at 4 °C, and the samples were stored at −80 °C for further analysis. Liver and epididymal adipose were collected, weighed, and frozen in liquid nitrogen for further analysis. Five livers were used for hepatic lipid content analysis, and the remaining livers (3–4) were used for PCR analysis.

### 2.3. Blood Glucose, Plasma, and Hepatic Lipid Analysis

Tail-vein blood glucose level was determined using a OneTouch Ultrameter (Life Scan Inc., Milpitas, CA, USA). Plasma lipoprotein cholesterol was determined according to our previous method [15,16]. Plasma triglyceride (TG) was determined using an enzyme colorimetric assay kit (Sekisui Diagnostics PEI Inc., Charlottetown, PE, Canada) according to the manufacturer’s instructions, and the absorbance was measured at 505 nm (Nanodrop 2000 C spectrophotometer, Thermo Scientific, Pleasanton, CA, USA). Liver lipids were determined as described previously [15]. Liver tissue from five mice from each group was used for hepatic lipid analysis and the residual liver tissue (*n* = 4) were saved for PCR analysis.

### 2.4. Glucose Tolerance Test (GTT)

The glucose tolerance test was administered after 6 weeks of diet treatment. After 5 h of fasting, mice were orally administered a 20% glucose solution (10 mL/kg body weight). Tail-vein blood glucose levels were determined at 0, 15, 30, 60, and 120 min using a OneTouch Ultrameter (Lifescan Inc., Milpitas, CA, USA). GTT curves were obtained by plotting glucose concentration versus time, and integrated glucose concentration over 120 min was calculated as the area under the curve (AUC).

### 2.5. Plasma Levels of Metabolic Biomarkers Relevant to Diabetes and Obesity

Plasma ghrelin, gastric inhibitory polypeptide (GIP), glucagon-like peptide-1 (GLP-1), insulin, leptin, resistin, and glucagon levels were analyzed using a mouse diabetes multiplex antibody assay kit (Bio-Plex Pro Mouse Diabetes Assay, Bio-Rad, Hercules, CA, USA) on the Bio-Plex 200 system (Bio-Rad, Hercules, CA, USA) according to the manufacturer’s instructions.

### 2.6. Plasma Levels of Inflammation Cytokines

Plasma interleukin-2 (IL-2), interleukin-4 (IL-4), interleukin-5 (IL-5), interleukin-10 (IL-10), interleukin-12 (IL-12), granulocyte-macrophage colony-stimulating factor (GM-CSF), interferon gamma (IFN-γ), and tumor necrosis factor alpha (TNF-α) levels were analyzed using a multiplex antibody kit following the manufacturer’s instructions (Bio-Plex Pro Mouse Cytokine Assay 8-plex, Bio-Rad) with the Bio-Plex 200 system (Bio-Rad).

### 2.7. Homeostatic Model Assessment of Insulin Resistance (HOMA-IR) Index Calculation

HOMA-IR was calculated from fasting blood glucose and plasma insulin concentrations. HOMA-IR index was calculated according to Equation (1) [17]. Insulin and plasma glucose concentrations after 16 h fasting were used to calculate the HOMA-IR index.

(1) $$\mathrm{HOMA}-\mathrm{IR}=\frac{\mathrm{Fasting}\;\mathrm{plasma}\;\mathrm{insulin}\;\left(\mathrm{mU}/L\right)\times \;\mathrm{Fasting}\;\mathrm{plasma}\;\mathrm{glucose}\;\left(\mathrm{mmol}/L\right)}{22.5}$$

### 2.8. Fecal Microbiome Analysis

Mice were placed in paper cups fecal pellets were immediately collected and, stored at −80 °C. DNA from feces was extracted using Qiagen DNeasy PowerSoil kits (Qiagen, Valencia, CA, USA) following the standard protocol. The V3–V4 domains of the 16S rRNA were amplified using primers 319F/806R (TCGTCGGCAGCGTCAGATGTGTATAAGAGACAG (spacer) GTACTCCTACGGGAGGCAGCAGT and [GTCTCGTGGGCTCGGAGATGTGTATAAGAGACAG (spacer) CCGGACTACNVGGGTWTCTAAT, respectively) containing an Illumina tag sequence, a variable-length spacer, a linker sequence, and the 16S target sequence. Each sample was barcoded with an Illumina P5 adapter sequence, a unique eight nucleotide (nt) barcode, and a partial matching sequence of the forward primer, as well as reverse primers with an Illumina P7 adapter sequence, unique 8 nt barcode, and a partial matching sequence of the reverse adapter. The final product was quantified on a Qubit 4.0 instrument using the dsDNA Broad Range DNA kit (Invitrogen, Carlsbad, CA, USA), and individual amplicons were pooled in equal amounts. The pooled library was cleaned with Ampure XP beads (Beckman Coulter, Brea, CA, USA), and bands of interest were further isolated by gel electrophoresis (Sage Science, Beverly, MA, USA). The library was quantified via qPCR then sequenced with 300 bp dual end sequencing with an Illumina MiSeq at the Genome Center DNA Technologies Core, UC Davis. The names of the repository/repositories and the accession numbers can be found at https://www.ncbi.nlm.nih.gov/ (accessed on 4 August 2021), PRJNA722496.

The Raw FASTQ files and adapter trimmings were demultiplexed with dbcAmplicons version 0.8.5 (https://github.com/msettles/dbcAmplicons (accessed on 4 August 2021)). Forward and reverse unmerged reads were imported into QIIME2 version 2020.2 (https://qiime2.org (accessed on 4 August 2021)), and sequence variants were determined by utilizing the DADA2 analysis pipeline. Singletons and chimeras were removed as part of the quality filtering process, and the remaining sequences were clustered into amplicon sequence variants (ASVs). The clustered sequences were then compared against the Silva 132 reference database which was used for taxonomic assignment, meeting 99% identity. 

Shannon’s index was calculated and displayed using the R program through rarefactions to indicate alpha-diversity. Beta-diversity was used to evaluate differences by both weighted and unweighted UniFrac methods. Subsequently, a principal coordinate analysis (PCoA) based on Bray–Curtis distance was performed with an iterative algorithm. An online LEfSe analysis was adopted to search for the biomarkers of different groups (http://huttenhower.sph.harvard.edu/galaxy (accessed on 4 August 2021)). Based on the LEfSe analysis, bacteria with *p*-values < 0.05 in LDA scores of 3.0 was plotted.

### 2.9. RT-PCR

RNA was extracted from livers and adipose tissues by TRIzol and an RNA purification kit (Invitrogen, Life Technologies, Carlsbad, CA, USA). All primers and probes for ddPCR were designed by Invitrogen (Invitrogen, Life Technologies, Carlsbad, CA, USA) as per MIQE guidelines [18]. cDNA was synthesized using a GeneAmp RNA PCR kit (Applied Biosystems, Foster City, CA, USA). Synthesized cDNA was diluted 10 times with dH2O, and 1 μL of diluted cDNA was used in each real-time RT-PCR using SYBR green supermix (Bio-Rad, Hercules, CA, USA) with an Mx3000P instrument (Agilent, Cedar Creek, TX, USA). Cycle conditions were as follows: 5 min at 95 °C and 94 °C for 30 s, followed by 60 °C for 1 min, and then 72 °C for 30 s. Primers were validated by PCR product sizes, and no primer dimers were observed in gel electrophoresis of PCR products. Primer amplification efficiency was over 90% for every RT-PCR assay. Differences in mRNA expression in liver and adipose tissues were calculated after normalization to β-actin or 36B4 mRNA expression using the 2^−∆∆Ct^ method [19]. The genes used in this study were Srebf1 (NCBI gene ID:78968), Cyp7a1 (NCBI gene ID:13122), Ppara (NCBI gene ID:19013), Cyp51 (NCBI gene ID:13121), Scd1 (NCBI gene ID:20249), Fasn (NCBI gene ID:14104), Slc2a4 (NCBI gene ID:20525), Adipoq (NCBI gene ID:11450), 36b4 (NCBI gene ID:11837), and β-actin (NCBI gene ID: 11461). Primers are shown in Supplementary Table S1.

### 2.10. Statistical Analysis

Results were expressed as the mean ± SEM. The significance of differences between treatments was analyzed by ANOVA, followed by Tukey–Kramer HSD tests, using 2016 SAS (version 9.4, SAS Inc., Cary, NV, USA). The significance level was set at *p* < 0.05.

## 3. Results and Discussion

### 3.1. Animal Metrics

**Figure 1:**
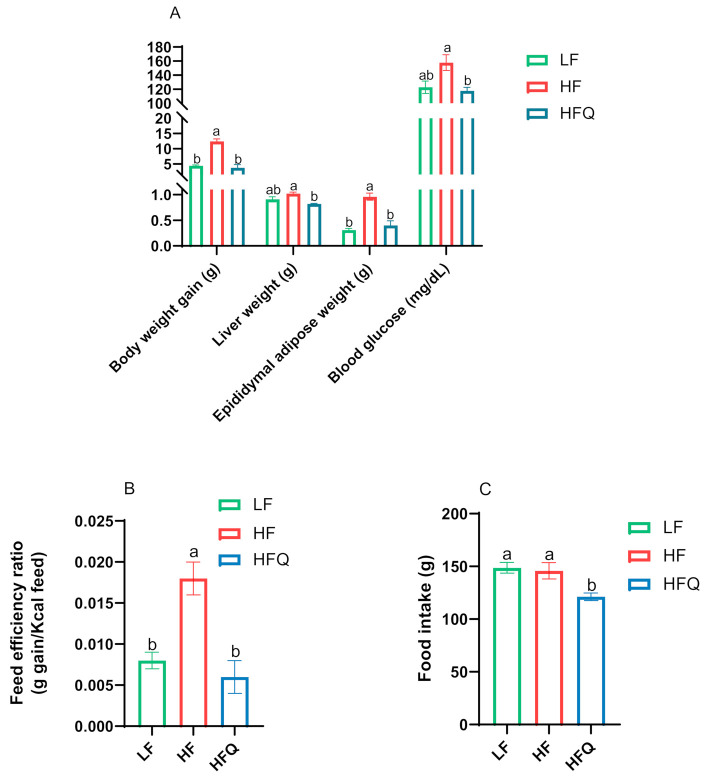
(**A**). Anthropometrics in mice fed HF, HFQ, and LF diets for 6 weeks. HF: high-fat control diet (46% kcal from fat, 16.5% kcal from protein, and 37.5% kcal from carbohydrate); HFQ: 0.05% quercetin in high-fat diet; LF: low-fat control diet (16% kcal from fat, 20% kcal from protein, and 64% kcal from carbohydrate) (**B**). Feed efficiency ratio of mice fed different diets. (**C**). Food intake of mice fed different diets. Values are means ± SEMs, *n* = 8/group. Bars with different letters were significantly different (*p* < 0.05).

Mice fed an HF diet had almost three times higher weight gain compared to mice on the LF diet, confirming HF diet-induced obesity (Figure 1A). Published reports of weight gain of diet-induced obese (DIO) mice fed quercetin have not been consistent. In our study, mice fed the HFQ diet gained 69.7% less weight (*p* < 0.05) and had a 66.7% lower feed efficiency ratio (g gain/calories intake, *p* < 0.05) than mice on the HF diet. Weight gain and feed efficiency (Figure 1B) of mice fed HFQ diet were similar to the LF group (*p* > 0.05). The food intake data are shown in Figure 1C. The total food intake of mice on the HFQ diet is less than the HF group. However, the feed efficiency ratio (g gain/g feed) was decreased significantly with quercetin supplementation. Quercetin supplementation might affect appetite. Porras and coworkers [20] reported a similar lower weight gain (77% of the control) and lower feed intake in C57BL/6J mice fed a 60% fat calorie diet containing 0.05% quercetin for 12 weeks. However, others have reported that quercetin supplementation of the HF diet fed to C57BL/6J mice did not result in differences in body weight. Kuipers and coworkers [21] reported no differences in body weight or feed consumption in the same mice model fed a 45% fat diet supplemented with 0.1% quercetin for 12 weeks. The differences in outcomes may be due to the lower percentage of fat calories, 46% vs. 53%, or the low solubility of quercetin. A previous study of isorhamnetin, an O-methylated quercetin glycoside abundant in onions, suggested that the glucose component in the quercetin glucoside affects its bioavailability [22]. The application of quercetin is also limited due to stability and solubility issues [23]. In this study, quercetin solubility and bioavailability were optimized by dissolving in alcohol to a molecular form before dispersing on the dry ingredients. The fat content of diet might influence the bioavailability and metabolism of quercetin [24]. 

Mice on the HFQ diet for 6 weeks had significantly (*p* < 0.05) lower liver and adipose weight, 19.6% and 58.3%, respectively, than those on the HF diet. The fasting blood glucose level of the mice on the HFQ diet was 25.4% lower than that of those on the HF diet. Final body weight, liver weight, adipose weight, and blood glucose levels of mice fed the HFQ and LF diets were not significantly different. This suggests that supplementation of quercetin in the HF diet has health-promoting effects in terms of preventing increases in in body, liver, and epididymal adipose tissue weights associated with mice on the HF diet. Previously, researchers reported that C57BL/6J mice fed a high-fat diet (39.9% energy from fat) supplemented with 0.05% quercetin had reduced body weight, blood glucose, insulin, cholesterol, TNF-α, and other markers of metabolic syndrome after 20 weeks of feeding but not after 8 weeks [7]. Since the present study did not first induce metabolic syndrome in mice followed by treatment with quercetin, this indicated that the supplementation with quercetin delays the development of obesity.

### 3.2. Plasma/Hepatic Lipid Content and Triglyceride (TG) Levels

**Figure 2:**
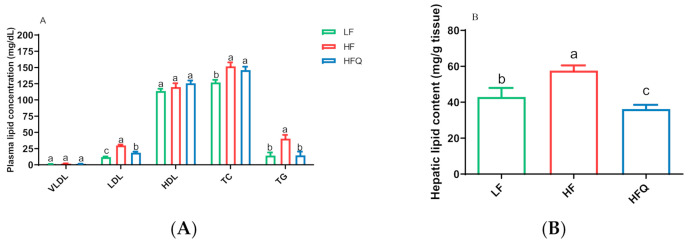
(**A**). Plasma lipoprotein cholesterol concentration and triglyceride level in mice fed a high-fat (HF) diet, high-fat diet containing 0.05% quercetin (HFQ), and low-fat (LF) diet for 6 weeks. VLDL: very-low-density lipoprotein; LDL: low-density lipoprotein; HDL: high-density lipoprotein; TC: total cholesterol; TG: triglyceride. Data are expressed as means ± SEMs, *n* = 8/group. Bars with different letters within the same plasma lipoprotein were significantly different (*p* < 0.05). (**B**). Hepatic total lipid content in mice fed with high-fat (HF) diet, high-fat diet containing 0.05% quercetin (HFQ), and low-fat (LF) diet for 6 weeks. Data are expressed as means ± SEMs, *n* = 5/group. Bars with different letters were significantly different (*p* < 0.05).

Quercetin supplementation of the HF diet lowered low-density lipoprotein (LDL) and plasma TG concentration by 37.6% and 62.9%, respectively, compared with the HF diet (*p* < 0.05) (Figure 2A). However, plasma very-low-density lipoprotein (VLDL) and high-density lipoprotein (HDL) cholesterol concentrations of mice fed the HFQ diet were not different from those on the HF diet (Figure 2A). The hepatic total lipid level of mice fed the HFQ diet was 35.5% lower than that of those on the HF diet (Figure 2B). The reduction in plasma TG and hepatic lipid levels was similar to that seen by Kobori and coworkers [7], who reported 27% lower plasma TG and about 30% lower hepatic lipids in C57BL/6J mice fed a Western diet (39.9% energy from fat) supplemented with 0.05% quercetin after 20 weeks but not at 4 or 8 weeks. The present study indicates that the HFQ diet may be responsible for the lowered total lipid content in liver after 6 weeks.

### 3.3. Glucose Tolerance Test (GTT) and Insulin Resistance

**Figure 3:**
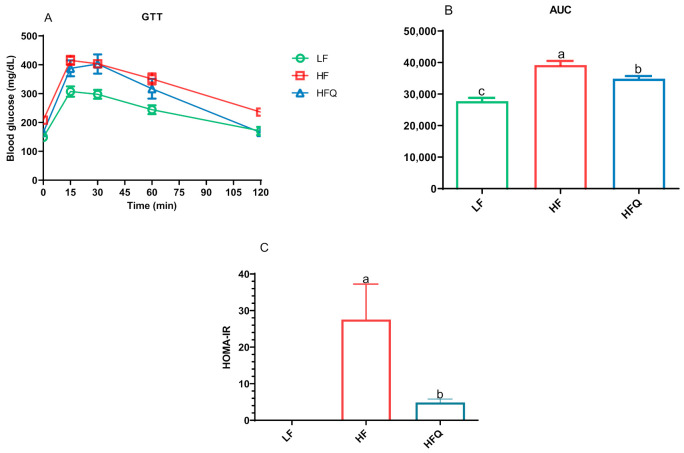
(**A**). Glucose tolerance in mice fed a high-fat (HF) diet, high-fat diet containing 0.05% quercetin (HFQ), and low-fat (LF) diet for 6 weeks. (**B**). Area under glucose tolerance test (GTT) curve values. (**C**). Homeostatic model assessment of insulin resistance (HOMA-IR) index of HF and HFQ diet-fed mice; the HOMA-IR index for the LF group was 0. Data are expressed as means ± SEMs, *n* = 8/group. Bars with different letters were significantly different (*p* < 0.05).

The oral glucose tolerance test (GTT) curve, the area under the curve (AUC), and the HOMA-IR index are shown in Figure 3A–C, respectively. Compared to the HF diet, HFQ significantly lowered the fasting blood glucose level (*p* < 0.05, Figure 1A). The blood glucose levels of the GTT for the HFQ-fed mice were lower at 15, 30, and 60 minutes but were not significantly different from HF. The HFQ diet significantly improved glucose metabolism (*p* < 0.05), lowered AUC by 10.9% compared to the HF diet. The HOMA-IR index of mice fed the HF diet was 82.3% higher than that of mice on the HFQ diet, suggesting that quercetin supplementation contributed to improved HF diet-induced IR. In the Figure 3C, HOMA-IR index of LF groups was 0, but not included in Figure 3C. In B6 mice fed an HF diet containing 0.4% quercetin for 26 weeks, it was reported that fasting blood glucose levels was lower but not significant [25]. Vessal and coworkers [26] reported that the glucose tolerance of STZ-induced diabetic rats returned to normal levels after intraperitoneal (ip) administration of quercetin for 10 days due to the regeneration of pancreatic islets. This experiment suggests that quercetin itself is bioactive as opposed to metabolites of gut bacterial metabolism. However, quercetin or its glycosides may be excreted through the bile, recirculated into the intestinal lumen, and made accessible to gut bacteria for metabolism into phenolic acids [27]. Kobori and coworkers [7] suggested that, in mice fed high-fat Western type diets, quercetin enables the recovery of cell functions in the liver and pancreas by reducing oxidative stress.

### 3.4. Plasma Biomarkers of Diabetes and Obesity

**Figure 4:**
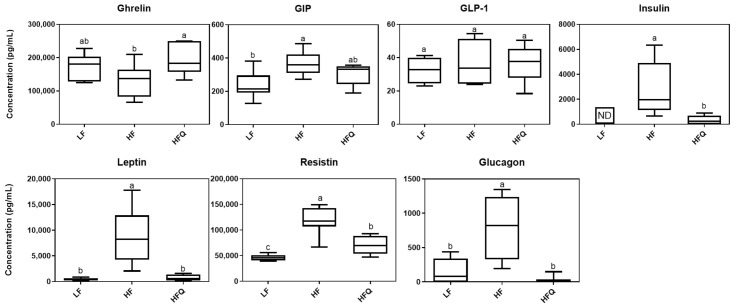
Boxplot of plasma biomarker concentrations related to diabetes and obesity, *n* = 8/group. ND = not detected. Top edge of the box, 75th percentile; bottom edge, 25th percentile; horizontal bar within box, median; top horizontal bar outside box, maximum concentration; bottom horizontal bar outside box, minimum concentration. Boxes with different letters were significantly different (*p* < 0.05). ND represents not detected.

Plasma ghrelin, GIP, GLP-1, insulin, leptin, resistin, and glucagon levels were analyzed using a multiplex immunoassay method (Figure 4). Mice fed the HFQ diet had a 34.5% higher ghrelin concentrations and 88%, 92%, 27%, and 97% lower insulin, leptin, resistin, and glucagon levels, respectively, than those on the HF diet. Plasma GIP and GLP-1 levels of HFQ diet fed mice were not significantly different from those on the HF (*p* > 0.05).

Ghrelin is an orexigenic hormone secreted mainly by the stomach preprandially and is responsible for regulating appetite and energy hemostasis. Ghrelin stimulates appetite; thus, our finding that the ghrelin level in mice fed the HFQ diet was higher but the feed intake and feed efficiency ratio were lower than in the HF diet-fed mice was unexpected. Ghrelin levels are higher before meals and lower between meals. In the current study, mice were sacrificed after a 16 h fast; therefore, high ghrelin levels in mice were expected. However, Moesgaard and coworkers [28] reported that the feeding status (fasting or non-fasting) does not affect ghrelin levels, and the reduced expression of ghrelin in obese C57BL/6J mice was due to decreasing numbers of ghrelin-producing cells. After gastric bypass, DIO mice had higher levels of ghrelin than mice without bypass, suggesting that lower feed intake may be associated with ghrelin levels [29]. In this study, ghrelin levels in mice fed LF diet tended to be higher, supporting the hypothesis that a HF diet reduces ghrelin-producing cells. Previous studies have not reported the effect of quercetin feeding with HF diets on the secretion of ghrelin.

Plasma insulin was lower in mice fed a HFQ diet compared to HF diet-fed mice. A high fasting plasma insulin level is an indicator of IR, and IR often precedes T2D. Plasma insulin and blood glucose levels of mice fed the HF diet were 83.3% and 25.4% higher (*p* < 0.05), respectively, than those on the 0.05% HFQ diet after 6 weeks. Kobori et al. [7] reported higher plasma insulin concentrations of C57BL/6J mice fed a Western diet (39.9% energy from fat) compared to the Western diet containing 0.05% quercetin after a 20 week feeding study but not at 8 weeks. However, the same researchers reported that, in STZ-induced diabetic BALB/c mice, low-fat (AIN93) diets containing 0.5% quercetin restored plasma insulin compared to STZ diabetic controls (*p* < 0.05) but did not reach the levels of the normal nondiabetic mice [30]. These results indicated that quercetin supplementation lowers insulin secretion compared to the HF diet.

Leptin is an adipokine that regulates energy balance by inhibiting hunger. In humans, obesity is also associated with higher serum leptin levels, indicating leptin resistance. In this study, serum leptin levels of mice fed the HFQ and LF diets were significantly (*p* < 0.05) lower compared to HF, suggesting normal leptin sensitivity (Figure 4). Few studies have reported the effect of quercetin intake on plasma leptin level. Hoek-van den Hil and coworkers [31] reported a lower body weight gain in C57bl/6JOlaHsd mice fed a HF diet containing 0.33% quercetin for 12 weeks. They reported that serum leptin and its adipose gene expression were lower compared to HF controls. In rats, Wein and coworkers [32] reported no differences in leptin levels between HF or 0.03% HFQ diets for 4 weeks, possibly due to the lower level of quercetin and the shorter feeding period.

Resistin is a proinflammatory adipokine related to insulin resistance and obesity. Zhang and coworkers [33] reported that quercetin (75 mg/kg/day) decreased serum resistin in a rat model of nonalcoholic fatty liver disease induced by a HF diet for 8 weeks. Studies of quercetin on plasma resistin are scarce, however, related flavonoids from herbs have shown effects on resistin concentration. The infusion of quercetin and *Ruta graveolens*, a traditional medicinal plant native to Europe, inhibited resistin expression in adipose tissue through downregulation of the resistin-encoding gene [34].

Glucagon is a hormone produced by the α cells of the pancreas that promotes glucose synthesis and fatty-acid oxidation in the liver. Glucagon’s action opposes that of insulin, and higher levels of glucagon in humans have been associated with insulin resistance [35]. The lower level of plasma glucagon in HFQ fed mice in this study supports the HOMA-IR index for increased insulin sensitivity [36]. To the best of our knowledge, the present study is the first to report the inhibitory effect of a HFQ diet on plasma resistin and improvement of HOMA-IR by reducing glucagon secretion.

### 3.5. Plasma Levels of Inflammatory Cytokines

There were no differences in all plasma cytokines, IL-2, IL-4, IL-5, IL-10, IL-12, GM-CSF, IFN-g, and TNF-α, between HFQ and HF or LF treatments analyzed using the multiplex immunoassay, as shown in the Supplementary Figure S1. However, despite this lack of difference in cytokine levels, several studies have found that quercetin decreases TNF-α. C57BL/6 mice fed quercetin at the same concentration (0.05% of diet) as this study but in a diet with higher fat content (60% energy from fat) for 9 weeks had a lower TNF-α level [37]. Kobori and coworkers [7] reported a decrease in TNF-α at 20 weeks but not at 8 weeks in C57BL/6J mice fed a diet with a similar fat content to the present study. The lack of a difference between treatments in our study may be due to a need for longer high-fat feeding time required to develop inflammation and the fat level of the diet.

### 3.6. Fecal Microbiota Analysis

**Figure 5:**
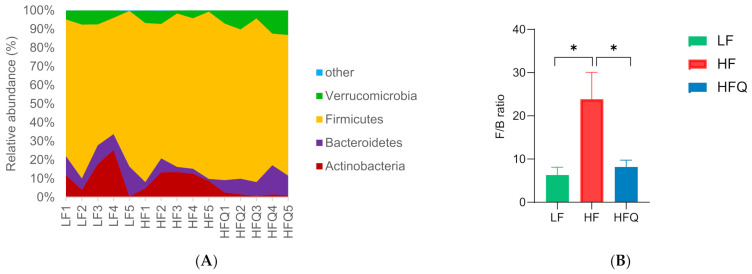

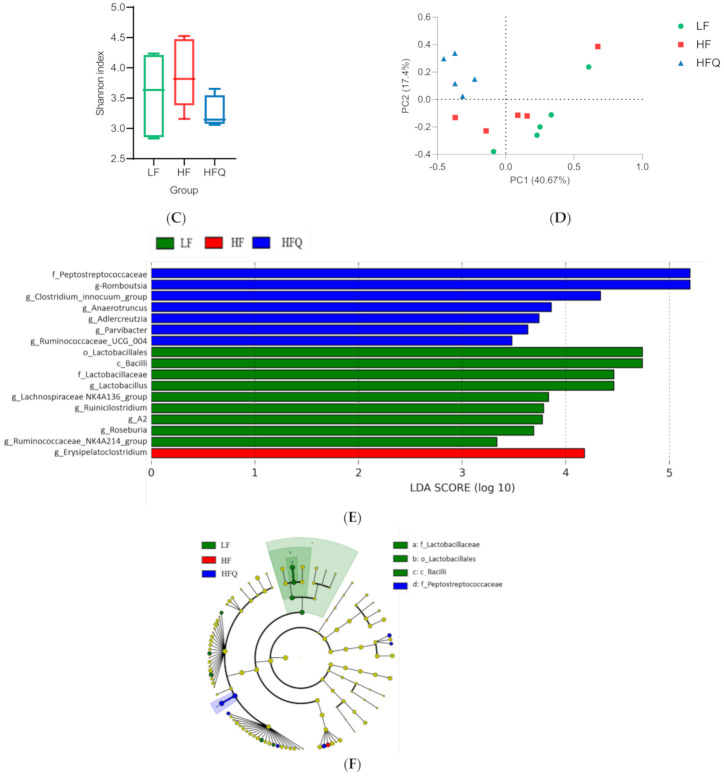
(**A**) Effect of 0.05% quercetin on the relative abundance of the four most abundant bacterial phyla (*n* = 5) in LF (low−fat), HF (high−fat), and HFQ (high−fat with 0.05% quercetin) diets. (**B**) Firmicutes/Bacteroidetes ratio of HF, HFQ, and LF diets for 6 weeks. (**C**). The alpha−diversity was assessed by calculating the Shannon index. (**D**) The beta−diversity was calculated by principal coordinate analysis (PCoA) for the visualization of pairwise community dissimilarity (Bray−Curtis index) of the microbial community. (**E**) Results of linear discriminative analysis (LDA). (**F**) Effect size (LefSe) analysis among three groups. Cardiogram showing differentially abundant taxonomic clades with an LDA score of 3.0 among groups with a *p*−value of 0.05; *n* = 5/group. An asterisk (*) indicates a significant difference.

Quercetin supplementation of the HF diet resulted in beneficial changes in the microbiome compared with HF. Quercetin lowered the relative abundance of the phyla Actinobacteria and Firmicutes (87.4% and 14.9%, respectively) but increased the abundance of Bacteroidetes (49.3%) (Figure 5A). The ratio of Firmicutes/Bacteroidetes (F/B) (Figure 5B) was significantly reduced by 65.6% by the HFQ diet (*p* < 0.05), and it was not different from LF diet. The ratio of Firmicutes to Bacteroidetes, F/B, is often cited as a marker of microbiota-associated obesity [38]. Firmicutes and Bacteroidetes account for 90% of the phyla in humans, and other phyla such as Actinobacteria are present at about 5–6%. Porras and coworkers [22] found that an HF diet induced nonalcoholic fatty liver disease in mice, which had an increased relative abundance of Bacteroidetes. However, Firmicutes were not significantly affected by 0.05% quercetin supplementation in the HF diet (*p* > 0.05). Turnbaugh and coworkers [39] claimed that the gut microbiota in ob/ob mice were more efficient at releasing calories from the diet than their lean siblings. The present study is the first to report that mice fed HF diets supplemented with quercetin significantly lowered the relative abundance of Firmicutes and increased the relative abundance of Bacteroidetes (*p* < 0.05). The alpha-diversity (Figure 5C) of the gut microbiota in the HFQ group was similar to that of the HF and LF groups, suggesting that quercetin supplementation did not affect the diversity of the gut microbiota. The results of the beta-diversity analysis illustrated that the taxonomic composition was distinctly different between the HF and LF groups (Figure 5D). To further characterize the changes to the gut microbiota, an LEfSe analysis was used to gain insight into the differences among the groups (Figure 5E,F). One significantly different class and one family were identified; Bacilli was higher in the LF group, and Peptostreptococcaceae was higher in the HFQ group. According to the LEfSe analysis, these abundant taxa could be considered as potential biomarkers (LDA score > 3.0, *p* < 0.05).

At the family level, the relative abundance of Akkermansiaceae, Bacteroidaceae, Eggerthellaceae, and Peptostreptococcaceae in the HFQ diet-fed mice was greater than that of HF diet alone. The HFQ diet significantly reduced the relative abundance of Atopobiaceae and Erysipelotrichaceae compared to the HF diet (*p* < 0.05). Erysipelotrichaceae has associated with diet-induced obesity [14]. Rabot [40] reported that diet-induced obese mice had higher levels of the Erysipelotrichaceae family. The Pearson correlation analysis between leptin and Erysipelotrichaceae suggested a strong correlation *r* = 0.99, *p* < 0.02. In contrast to Erysipelotrichaceae, the relative abundance of the Bacteroidaceae family in HFQ diet-fed mice was higher in the present study and supports the work of Etxeberria et al. who reported an increasing relative abundance of the Bacteroidaceae family induced by the HFQ diet, although the changes were not statistically significant (*p* > 0.05) [14]. The enrichment of the Bacteroidaceae family was found to be negatively associated with HF diets in mice. Thus, the result from the current investigation suggested that the HFQ diet enrichment of the Bacteroidaceae family may have contributed to a gut microbiome with a higher level of Bacteroidaceae family. Tan et al. (2018) reported that quercetin fed for 12 weeks had a similar effect on body weight, metabolic features, and the gut microbiome, particularly when given with a soluble fiber [41]. It was reported that quercetin was metabolized in the gut after administration, and its methylated metabolite isorhamnetin was the dominant form in the serum; however, the metabolites of quercetin in the gut remained unclear [42]. Moreover, the authors also evaluated the short-chain fatty acids (SCFAs) in the fecal samples, and results suggested that quercetin-treated mice exhibited significantly (*p* < 0.05) higher levels of butyrate in their feces compared with the antibiotic-treated mice [42]. Moreover, quercetin supplementation did not seem to modify the intestinal barrier permeability-associated markers TJP-1, TJP-2, and Ocln gene [12]. Jin et al. reported that quercetin increased the expression of ZO-1 in rats treated with quercetin [43]. These two studies indicated that quercetin might affect the tight junction protein level of ZO-1but not Ocln. Weight gain was reported to be accompanied by the release of inflammatory cytokines from adipose tissues in response to lipopolysaccharides (LPS), cell-wall fragments of Gram-negative bacteria that pass from the intestinal lumen through the intestinal wall [44]. While Gram-negative bacteria are the sources of LPS and initiator of inflammation, other commensal and probiotic bacteria were shown to prevent or reduce the severity of diabetes and other metabolic diseases [45]. Correlation between glucagon and Peptostreptococcaceae was strong (*r* = 0.99, *p* < 0.04). The present study is the first to report that the relative abundance of Atopobiaceae, Eggerthellaceae, and Peptostreptococcaceae families was significantly affected by the HFQ diet compared to the HF fed mice.

At the genus level, the relative abundances of *Akkermansia*, *Bacteroides*, *Marvinbryantia*, and *Romboutsia* genera were significantly increased (64.2%, 67.9%, 65.1%, and 75.2%, respectively) by the HFQ diet compared to the HF alone. There have been few reports of the presence of *Romboutsia* in feces, but a recent report suggested that this genus was indicative of a healthy status of patients [46]. The relative abundance of *Blautia*, *Clostridium sensu stricto 1*, *Erysipelatoclostridium*, *Lactobacillus*, and *Turicibacter* genera was lowered by the HFQ diet compared to the HF diet, but this did not reach statistical significance. *Akkermansia muciniphila* is one of the most abundant species in the intestine, which has been found to be lower in obesity and lowered in individuals treated with metformin [47]. *Akkermansia* enrichment was inversely correlated with obesity [14]. We observed an increase by 64.2% in the relative abundance of *Akkermansia* in the feces of HFQ-fed mice, as well as a significant increase by 68.0% in the relative abundances of *Bacteroides*, *Marvinbryantia*, and *Romboutsia* genera in feces of the HFQ-fed mice compared to the HF diet. More propionate was produced by *Bacteroides*, and the lipid synthesis from acetate was inhibited by propionate [48]. The increased abundance of *Bacteroides* may contribute to weight loss via propionate inhibition of lipid synthesis. In addition, a significantly greater relative abundance of *Marvinbryantia* genus was observed in the feces of HFQ-fed mice. The relative abundance of *Marvinbryantia* was positively correlated with body weight [49]. However, the mechanism is unknown. The current results show that a HFQ diet reshapes the gut microbiome of mice in the HF diet group withtaxa associated with a lean phenotype and less metabolic dysfunction.

### 3.7. RT-PCR Analysis

**Figure 6:**
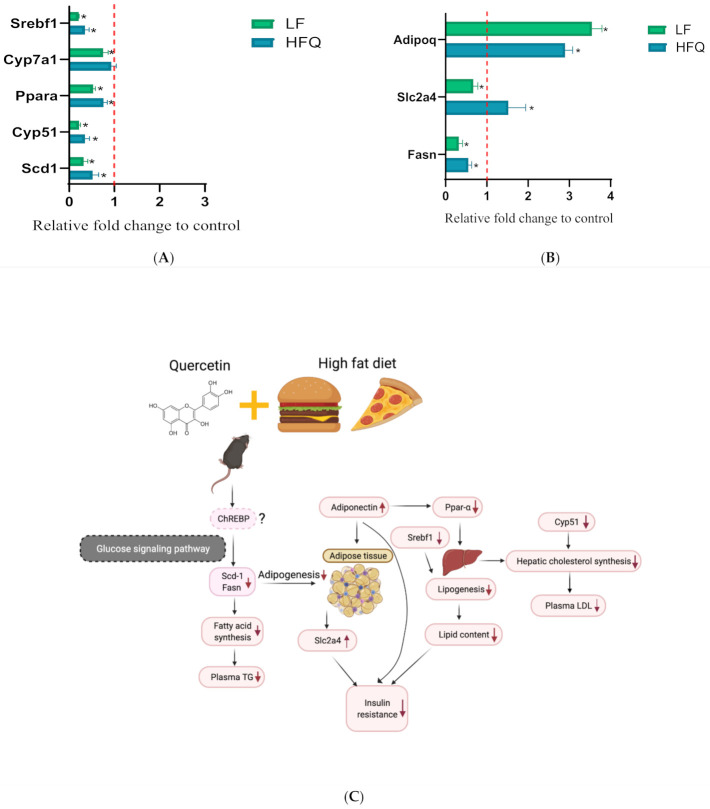
(**A**) Relative hepatic gene expression of *Srebf1*, *Cyp7a1*, *Ppara*, *Cyp51*, and *Scd1* in mice fed an HFQ diet compared to the HF diet (red dotted line), with the LF diet group shown as a reference. (**B**) Relative expression of *Fasn*, *Slc2a4*, and *Adipoq* genes in epididymal adipose tissue of mice fed an HFQ diet compared to the HF diet, with the LF diet group shown as a reference. Data are expressed as the mean ± SE, *n* = 4/group. Differences in mRNA expression for livers and adipose tissue were calculated after normalizing to 35B4 mRNA expression. An asterisk (*) indicates a significant difference (*p* < 0.05) compared to the HF diet. The dotted line (x = 1) represents the expression of control gene. (**C**) Proposed mechanism responsible for ameliorating insulin resistance in mice fed an HF diet supplemented with quercetin, involving the glucose signaling pathway, and ChREBP (dashes outline), as extrapolated from the literature [54].

Plasma TG, LDL and total hepatic lipid contents, fasting glucose, GTT, and HOMA-IR were significantly lower in mice fed HFQ diets compared to HF diet-fed mice. We, therefore, compared the expression of selected genes for fat and glucose metabolic pathways in liver and adipose tissue (Figure 6A,B, respectively). The mRNA levels of hepatic genes for major enzymes for cholesterol and bile acid synthesis, *Cyp51* (cytochrome P450, family 51, encoding lanosterol 14α-demethylase) was 0.35-fold lower than the HF diet, but the expression of *Cyp7a1* (cytochrome P450, family 7, subfamily a, polypeptide 1, encoding cholesterol 7-alpha-monooxygenase) was not changed (0.93 compared to HF). Lanosterol demethylase is considered to be the first committed step for the synthesis of sterols from lanosterol. *Cyp7a1* encodes the gene for cholesterol 7-alpha hydroxylase, the enzyme responsible for the rate-limiting step of bile acid synthesis. These results suggest that the observed lower LDL cholesterol was due to reduced hepatic cholesterol synthesis rather than excretion of bile acids. Fatty-acid metabolism-related genes, *Scd1* (stearoyl-coenzyme A desaturase 1) and *Srebf1* (sterol regulatory element binding transcription factor 1), were reduced by 0.52 and 0.35-fold, respectively. *Srebf1* is a gene regulated by insulin and codes for transcription factor that increase glycolysis and lipogenesis. Its reduced expression relative to HF suggests that the effect may have been due to lower plasma insulin. *Scd1* catalyzes the synthesis of monounsaturated fatty acids that serve as substrates for the synthesis and storage of triglycerides. The relative expression of *Ppara* (peroxisome proliferator activated receptor alpha), a nuclear transcription factor regulating hepatic fat metabolism, was 0.75 of the control. The lower expression of *Pparα* and *Scd1* suggests a lower uptake and oxidation of fatty acids and may be related to the lower liver TG levels (Figure 2B).

In adipose, fatty-acid synthase (Fasn) is a multienzyme protein that metabolizes acetyl- and malonyl-CoA derived from glucose into fatty acids. The downregulation of the *Fasn* gene may be related to the lower liver lipid content (Figure 2B and Figure 6B). *Slc2a4* (solute carrier family 2, facilitated glucose transporter member 4, also known as *Glut4*) is an insulin-regulated glucose transporter, and downregulation of Slc2a4 in adipose tissue indicated less glucose intake and, therefore, less fat storage in adipocytes in mice fed an LF diet. However, overexpression of Slc2a4 increases insulin sensitivity and glucose tolerance in obese mice [50]. Slc2a4 was significantly increased (Figure 6B) and supports the lower HOMA-IR and improved insulin sensitivity by the HFQ diet compared to the HF diet alone. Adiponectin is a protein hormone coded by the *Adipoq* gene by adipocytes which regulates glucose level and fatty-acid oxidation. *Adipoq* expression was almost three times higher than HF control (Figure 6B), confirming the plasma multiplex immunoassay results. Higher adiponectin levels are also associated with improved insulin sensitivity, as indicated by lower HOMA-IR. Decreased *Adipoq* is related to the development of IR [51]. The proposed mechanism responsible for ameliorating insulin resistance in mice fed an HF diet supplemented with quercetin is shown in Figure 6C. The increased level of *Akkermansia* was related to reduced insulin resistance [52]. The *Lactobacillus* level was reported to be positively associated with weight loss [53]. Gut microbiome changes are associated with insulin resistance; however, the mechanism behind the association remains unclear.

## 4. Summary

The present study demonstrated that chronic quercetin supplementation for 6 weeks significantly lowered plasma triglyceride level, decreased body weight gain, reduced liver fat accumulation, ameliorated insulin resistance, and decreased F/B ratio (associated with lean phenotype) of HF diet-induced obese mice. Plasma ghrelin, leptin, resistin, and glucagon changes were associated with improvements in metabolic health. The HFQ diet attenuated the increase in Firmicutes/Bacteroidetes ratio and modulated gut bacteria composition at both family and genus levels. Furthermore, chronic supplementation of quercetin resulted in a significant alternation in genera related to obesity (*Bacteroides* and *Akkermansia*). The present study suggested that chronic quercetin supplementation prevented the increase of many biomarkers of obesity-related metabolic dysfunction, and microbiota associated with lean phenotypes and healthy metabolic profiles were restored by quercetin supplementation.

## Figures and Tables

**Table 1:** Diet composition (grams).

Ingredient	High Fat Diet (HF)	High Fat Diet with Quercetin (HFQ)	Low Fat Diet (LF)
Lard fat	225.0	225.0	63.0
Soybean oil	25.0	25.0	7.0
Cholesterol	0.8	0.8	0.8
Cellulose	50.0	49.5	50.0
0.05% quercetin	-	0.5	-
Casein	200.0	200.0	200.0
Corn starch	148.2	148.2	528.2
Sucrose	300.0	300.0	100.0
Cystine	3.0	3.0	3.0
Choline bitartrate	3.0	3.0	3.0
Mineral mix	35.0	35.0	35.0
Vitamin mix	10.0	10.0	10.0
Total weight	1000.0	1000.0	1000.0
Calories/kg	4850.0	4850.0	3950.0

## Data Availability

Data are contained within the article.

## Supplementary Materials

The following are available online at https://www.mdpi.com/article/10.3390/antiox10081251/s1, Figure S1: Concentrations of plasma inflammatory cytokines, *n* = 8/group. Top edge of the box, 75th percentile; bottom edge, 25th percentile; horizontal bar within box, median; top horizontal bar outside box, maximum concentration; bottom horizontal bar outside box, minimum concentration. Boxes with different letters were significantly different (*p* < 0.05)., Table S1: gene primers used in the PCR assay.
