# Supplementary material for: Quercetin Ameliorates Insulin Resistance and Restores Gut Microbiome in Mice on High-Fat Diets
Source: Antioxidants (Basel). 2021 Aug 5;10(8):1251. doi: 10.3390/antiox10081251 (PMC8389224; doi:10.3390/antiox10081251)
Supplement: Supplementary file 1 [file antioxidants-10-01251-s001.zip › antioxidants-1319134-supplementary.pdf]

Supplementary material

Table S1. Gene primers used in the PCR assay

Gene(NCBI No.)	Primer direction	Primer (5′ → 3′)
Srebf1 (78968)	forward	CATGGATTGCACATTTGAAGACA
	reverse	CAGAGAAGCAGAAGAGAAGC
Cyp7a1 (13122)	forward	ACAGCTAAGGAGGACTTCACTCT
	reverse	TTCATCAAGGTACCGGTCGTATT
Ppara (19013)	forward	CTCCACCTGCAGAGCAACCA
	reverse	CGTCAGACTCGGTCTTCTTGAT
Cyp51 (13121)	forward	GAGAGAAGTTTGCCTATGTGCC
	reverse	TGTAACGGATTACTGGGTTTTCT
Scd1 (20249)	forward	AGGCTACATTGAGGTCCTGGT
	reverse	AGGGAAGGAGTGAGACTGAGC
Fasn (14104)	forward	GCAAAGTCCTTGTCCAGGTA
	reverse	TGCTTGGCTTGGTAGCCG
Slc2a4 (20528)	forward	TTCTATTTGCCGTCCTCCTGCTT
	reverse	TCATTCTCATCTGGCCCTAAGTAT
Adipoq (11450)	forward	GGAGAGAAGGGAGAGAAAGGAG
	reverse	GAACGCTGAGCGATACACATAA
36b4 (11837)	forward	TCTAGGACCCGAGAAGACCTC
	reverse	GTTGTCAAACACCTGCTGGAT

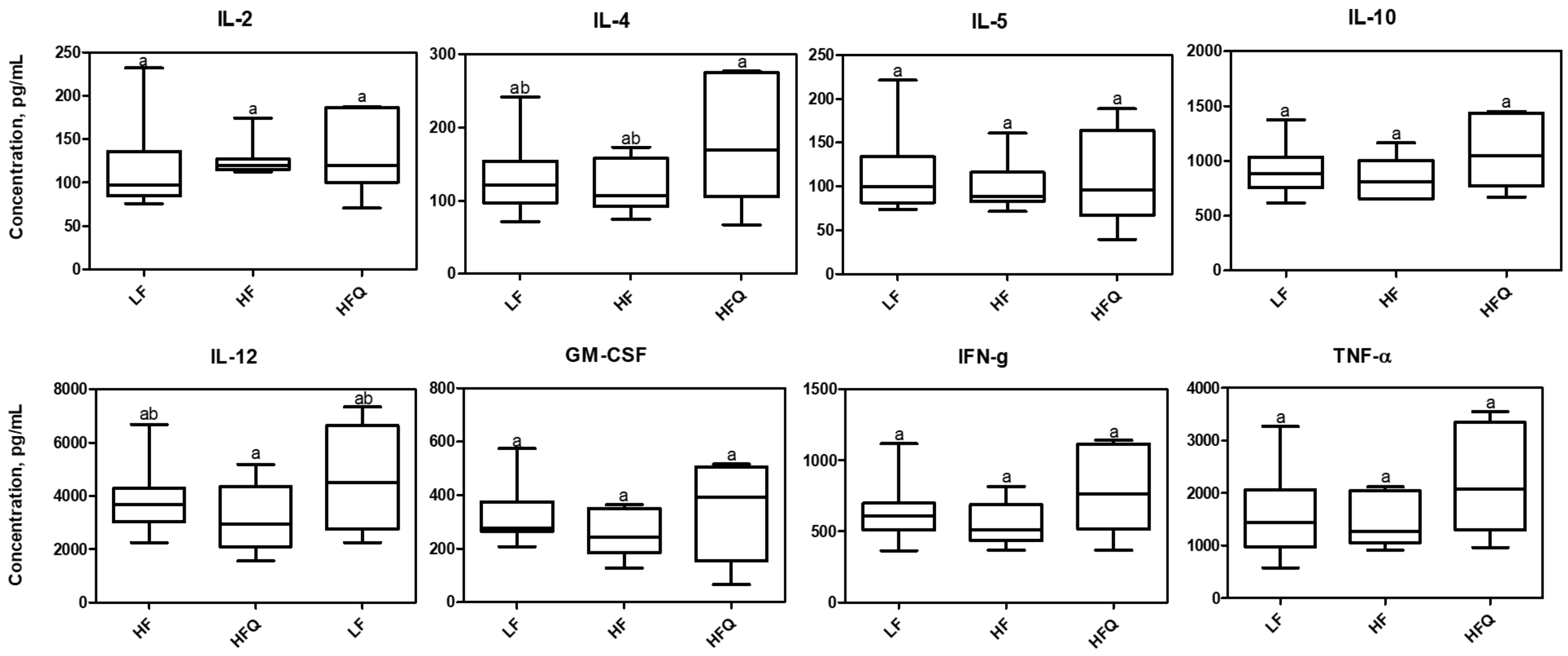

Figure S1. Concentrations of plasma inflammatory cytokines, n = 8/group. Top edge of the box, 75th percentile; bottom edge, 25th percentile; horizontal bar within box, median; top horizontal bar outside box, maximum concentration; bottom horizontal bar outside box, minimum concentration. Boxes with different letters were significantly different (p <0.05).
